# A Green Process for Effective Direct Recycling and Reuse of Graphite from End‐of‐Life Li‐Ion Batteries Black Mass

**DOI:** 10.1002/cssc.202500550

**Published:** 2025-07-08

**Authors:** Lorenzo De Vita, Daniele Callegari, Andrea Bianchi, Cristina Tealdi, Noemi Zucca, Pietro Galinetto, Marcello Colledani, Eliana Quartarone

**Affiliations:** ^1^ Department of Chemistry University of Pavia Via Taramelli 12 27100 Pavia Italy; ^2^ National Interuniversity Consortium of Materials Science and Technology INSTM Via Giusti 9 50121 Florence Italy; ^3^ Department of Physics University of Pavia Via Bassi 6 27100 Pavia Italy; ^4^ Department of Mechanics Polytechnic University of Milan Via Privata G. La Masa 1 20156 Milan Italy

**Keywords:** closed loops, direct recycling, froth flotations, graphites, lithium‐ion batteries

## Abstract

The demand for lithium‐ion batteries (LIBs) is posing challenges in the management of end‐of‐life (EoL) systems and supply of critical raw materials. Such challenges can be addressed by recycling EoL LIBs through sustainable processes, involving metallurgy to recover higher‐value metals like Co, Ni, and Li. Pretreatment strategies allowing to reclaim other valuable materials—such as graphite, binders, and electrolytes—are also crucial to enhance the overall recycling efficiency. Despite the strategic relevance of graphite in the battery supply chain, its recovery and reuse remain poorly explored. Herein, a sustainable closed‐loop approach for the reclamation and reuse of graphite from EoL LIBs black mass is proposed, exploiting a low‐impact froth flotation based on green chemicals. The recovered graphite is purified through a mild chemical leaching by natural organic acids and thermally treated to restore its microstructure from damages induced by the aging phenomenon. The regenerated material is characterized by multi‐sample technique approach, demonstrating high separation efficiency (>96% yield) and purity (>99.6%). The direct recycling process is validated by reusing the reclaimed graphite as secondary anode active material in new cells, showing functional performance comparable to those of the commercially available material.

## Graphical abstract

A sustainable closed‐loop approach for reclamation and reuse of graphite from end‐of‐life (EoL) lithium‐ion batteries (LIBs) black mass is proposed. Low‐impact froth flotation with green chemicals, followed by mild chemical purification and thermal treatment, is exploited. High‐quality graphite is obtained. The direct recycling is validated through a closed‐loop strategy from EoL to new cells, reusing the recovered material as regenerated anode.

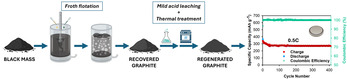

## 1 Introduction

Batteries are a key‐enabling technology to secure the energy transition.^[^ ^1^ ^]^ As the need for batteries surges, the demand for critical raw materials included in them (e.g., lithium, nickel, cobalt, copper, graphite, manganese, and phosphorus) will increase accordingly. High level of circularity, sustainability, and resource efficiency must be fostered to ensure a more resilient supply chain while addressing environmental issue and to develop additional sources for strategic raw materials necessary for the battery production.^[^ ^2^ ^]^ This objective can be achieved though circular economy process chains by expanding the recycling alternatives, for example, unlocking closed‐loop approaches and symbiosis with the other wastes.

The closed‐loop recycling value chain recovers important materials from the post‐use batteries and integrates them as secondary raw materials in the remanufacturing of new batteries. This approach contributes to decrease the dependency on primary battery materials sourcing and bring environmental benefits thanks to the lower secondary emissions.^[^ ^3^, ^4^ ^]^

The EU Battery Regulation gives clear indications on how to properly manage recycling and reuse of the growing number of end‐of‐life (EoL) batteries, mandating higher recovery targets for the critical elements and recycled contents.^[^ ^5^ ^]^ However, currently, only Li and the transition metals, such as Ni and Co, are considered in most governmental regulations, while inherently less valuable graphite is now excluded due to notoriously challenging cost‐effective recovery. Despite its lower value compared to the metals inside batteries, recycling graphite could produce economic benefits such as 1) enhancing the efficiency and profitability of the recovery technologies and 2) potentially reducing the carbon emissions associated with battery production by preventing that huge amount of graphite, sourced by spent batteries and manufacturing scraps, could be wasted and landfilled.^[^ ^6^ ^]^

To guarantee a worthwhile recycling process, the recovered graphite must be a battery‐grade product. To achieve that, efficient, scalable, and sustainable methods to separate graphite from the other battery materials (including black mass (BM)) are still needed.^[^ ^2^ ^]^

The existing recovery technologies are mostly employed for metals and trade‐off graphite. They are pyrometallurgy and hydrometallurgy, both considered “large‐loop” processes as they involve numerous continuous steps.^[^ ^3^ ^]^ Pyrometallurgy uses high temperature to smelt the BM and obtain alloys of Co, Ni (and eventually Cu, Fe) that can be further refined by hydrometallurgical routes. Graphite from the anode acts as reducing agent; burned under oxygen atmosphere, it helps to maintain the process temperature. Hydrometallurgy uses strong mineral acids and reducing agents to leach the metal ions from the BM that are subsequently precipitated in the salt form (typically metal sulfates for Ni, Co, and Mn). In order to obtain high recovery rates, graphite, carbon‐based residues (including the binders), and other feedstock impurities must be removed by pyrolysis before the chemical treatment.^[^ ^2^, ^3^ ^]^ The state‐of‐the‐art (SoA) pyro‐ and hydrometallurgy appear to hold disadvantages, such as high energy consumption, harsh conditions, and generation of toxic by‐products with consequent environmental concerns.^[^ ^7^ ^]^

Direct recycling is an alternative emerging sustainable process to recover the cathode active material (CAM) preserving its structure and reducing the need of raw materials/precursors synthesis, with advantages in terms of upcycling and reduced carbon footprint. It also allows the separation of graphite anode that can be further treated to be reused in a new battery.^[^ ^8^, ^9^ ^]^ This means to restore the pristine structure and morphology, to remove all the internal metallic impurities, including the solid electrolyte interphase (SEI), which is formed upon galvanostatic cycling.

Several articles well review the wide spectrum of separation methods explored to reuse graphite as battery‐grade material in a new cell or as lower‐value product in secondary applications (for example adsorbents, reductants). A number of approaches have been developed depending on how the EoL batteries have been previously treated.^[^ ^10^, ^11^, ^12^, ^13^ ^]^ In case of disassembly and separation of each single cell component (namely electrodes and separators), graphite can be recovered from the current collector by means of delamination and washing steps, thermal treatments, or even hybrid (both chemical and thermal) processes, electrochemical methods, usually followed by purification by acid leaching or supercritical fluid technology.^[^ ^10^, ^11^, ^12^, ^13^, ^14^, ^15^, ^16^, ^17^ ^]^ In case of BM, obtained via mechanical pretreatment (e.g., crushing and shredding), froth flotation is suitable to separate graphite from the cathode fraction.^[^ ^13^, ^18^, ^19^, ^20^ ^]^ This route advantageously results in higher rate of precious metallic fraction that can undergo metallurgy and in the feasibility of direct separation and reuse.

Flotation is commonly used to successfully separate minerals in the mining industry. It exploits the difference in wettability between the hydrophilic component (i.e., cathode) and the hydrophobic one (anode). and it is based on the liquid surface tension allowing the hydrophobic particles to be attached to air bubbles and then floated out.^[^ ^21^ ^]^ In order to enhance the wettability contrast, which is often limited, several types of surfactants are used in very small amounts during the process, namely the collector, to enhance hydrophobicity of the material to be floated, and a frother, to stabilize the air/solution interface, improving the efficiency of the separation.^[^ ^21^, ^22^ ^]^ The frother is the most crucial active agent, playing a key role in controlling important parameters for the achievement of high flotation efficiency and the improvement of the mineral properties and grade, such as the bubble size, the coalescence phenomena, and the stability and mobility of the froth layer. Ideally, the frother acts at the air/solution interface and should not affect the graphite surface. A huge number of chemical frothers have been discussed and reviewed in literature, ranging from alcohols (aliphatic, cyclic, and aromatic) to alkoxy paraffins and polyglycols.^[^ ^23^ ^]^ Widely used systems in the flotation of graphite are kerosene and methyl isobutyl carbinol (MIBC), respectively, as frothing agent and collector, which have been demonstrated to favor an efficient separation.^[^ ^24^ ^]^ Despite the intrinsic simplicity of this technology, the application of froth flotation to recover graphite from BM still deserves deep investigation, since it can be affected by several aspects, such as the graphite morphology, the nature of the SEI, and the type of frother and collectors.^[^ ^21^, ^25^ ^]^

Despite the urgency for the development of sustainable graphite recycling routes, limited efforts have been done so far to the quantification of the environmental impacts of such technologies, including flotation. The life cycle assessment (LCA) and life cycle inventory (LCI) still need to be implemented; however, a recent study with a specific focus on the global warming potential impact (GWP) reports values ranging from 0.53 to 9.76 kg·CO_2_ equiv.·kg graphite^−1^, strongly suggesting that graphite reclamation approaches (especially those based on flotation) are environmentally competitive with virgin graphite production (GWP in the range of 1–5 kg·CO_2_ equiv.·kg graphite^−1^).^[^ ^26^ ^]^

This work reports on the potential of a green process to directly recover and regenerate high‐quality graphite from an industrial EoL lithium‐ion batteries (LIBs) BM and its reuse as anode active material (AAM) in new cells. The proposed solution is based on the sole use of only green chemicals in the whole recycling concept, aiming to potentially reduce the overall process carbon footprint. The method is a two‐step route: 1) froth flotation based on paraffin as collector and pine oil as alternative frother to the conventional kerosene and/or MIBC. Contrary to kerosene, for which massive dosage is usually required to obtain decent flotation, with consequent severe environmental concerns,^[^ ^27^ ^]^ pine oil is here preferred due to several advantages in terms of good balance between process sustainability and flotation performances, such as low cost, flexibility of application (even without any additional collector), bio‐alternative to the petrochemically derived oils, capability to form stable foam, and high grade of flotation concentrates.^[^ ^28^ ^]^ 2) Graphite purification by means of natural organic acids (e.g., citric acid) though milder leaching in place of mineral acids and related harsh conditions.

The output of the recycling process was high‐quality graphite that has been characterized by a multitechnique approach (X‐ray diffraction (XRD), Raman spectroscopy, SEM–EDS, and ICP–OES). The process potential was underlined by comparing the graphite functional properties as anode in half cell with a commercial benchmark in terms of cycling stability, differential capacity plots, and initial Coulombic efficiency.

## 2 Experimental Section

### 2.1 Black Mass (BM) Production

Modules of EoL industrial Li‐ion batteries were pretreated at the CircEV facility hosted at the Polytechnic University of Milan (Italy). First, they were disassembled and discharged. After the thermal removal of the electrolyte solvents, the cells were mechanically treated by means of shredding and subsequent sieving to concentrate the valuable fraction, separating both the metallic and plastic components. The produced BM, with particle size <200 μm, was then characterized to identify the cell chemistry, quantify impurities, and evaluate the particle morphology. This fraction underwent thermal treatment at 470 °C for 2 h under air to remove the polymeric binders.

### 2.2 Froth Flotation

**Figure 1:**
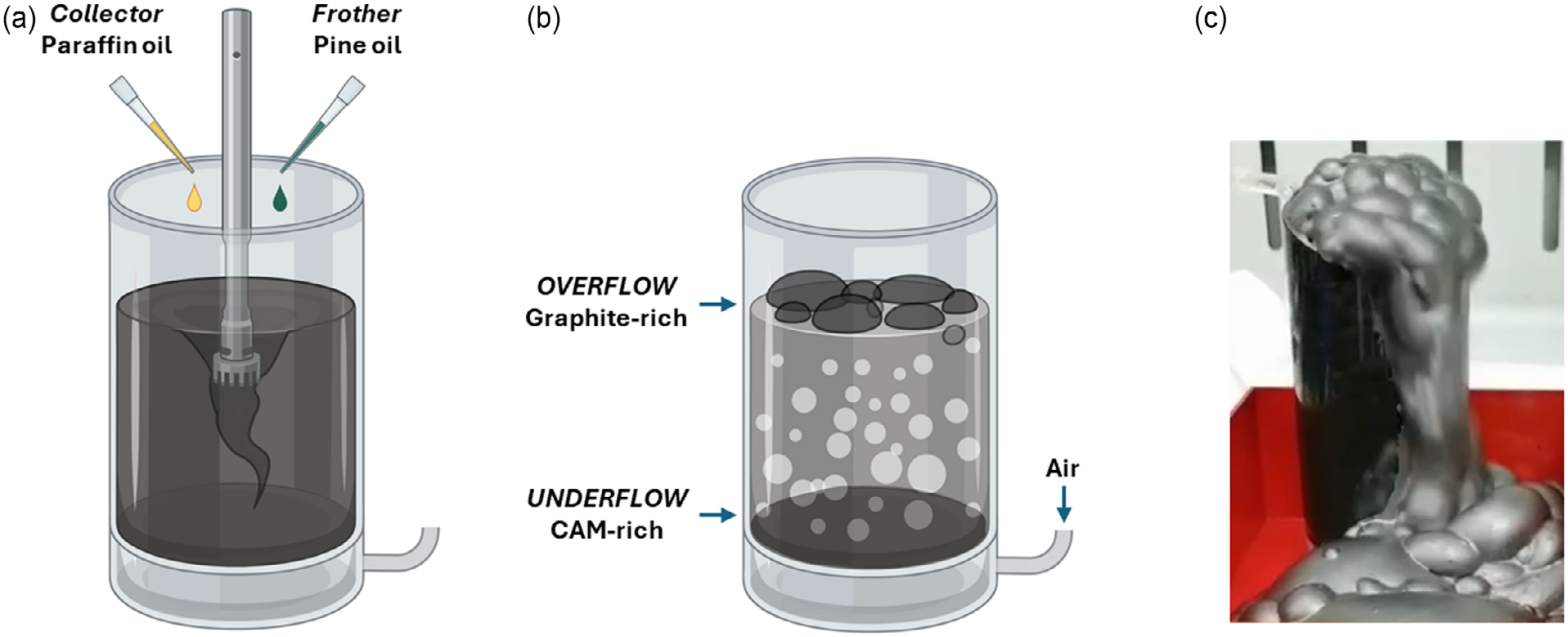
Scheme of the froth flotation procedure. a) Attritioning step with the addition of collector and frother additives; b) flotation step with air bubbling promoting the separation of an over‐ and an underflow. Created in BioRender, De Vita, L. (2025) https://BioRender.com/e43c712. c) Picture of the collected graphite‐rich froth.

The froth flotation procedure was performed in a customized 400 mL flotation cell, as pictured in **Figure** **1**. In each experiment, 10 g of BM were dispersed in 200 mL of deionized water (solid loading 5%) by means of an Ultra‐Turrax T‐25 (IKA) high‐shear mixer at 6500 rpm for 5 min to better disaggregate the particles by removing the residual binder (attritioning step).^[^ ^24^ ^]^

Then, the collector (paraffin oil, dose: 300 g t^−1^, Merck) and the frother (pine oil, dose: 300 g t^−1^, Merck) were added and properly dispersed by stirring for further 5 min (Figure 1a). Air was inlet in the cell at 10 L m^*−*1^ in to start flotation, as illustrated in Figure 1b. The froth overflowing from the vessel (see Figure 1c) was collected and the tailing fraction (richer in CAM) filtered and recovered at the end of the experiment. The graphite fraction was then dried at 80 °C under vacuum for 18 h and finally weighed to estimate the flotation yield. After flotation, the product was chemically purified by treatment with citric acid (0.5 M) at 75 °C for 2 h to remove the CAMs impurities, then washed with pure distilled water, filtered, and finally dried at 80 °C for 18 h. Purified graphite was then thermally treated in a tube furnace at 900 °C for 2 h under Ar flow.

### 2.3 Graphite Anode Preparation and Cell Assembly

The anode slurry was prepared by combining 93.2 wt% of recovered graphite, 2.5 wt% of conductive carbon black (Imerys, Super C65), 1.8 wt% of sodium‐carboxymethyl cellulose (Na‐CMC, Sigma‐Aldrich), and 2.5 wt% of styrene‐butadiene rubber (SBR, MTI Corp.), dispersed in distilled water (DiW). The solid content of the slurry was kept ≈22 wt%. The recovered graphite and carbon powders were previously mixed in zirconia jars by a planetary ball miller at 100 rpm for 10 min twice, with a rest period of 5 min. After that, the powder mixture was dispersed in DiW. Then, the polymeric binder was added and mixed to obtain the slurry, which was cast on a copper foil using a doctor blade with 300 μm wet thickness and then dried under vacuum at 80 °C. Subsequently, the manufactured electrode was subjected to a calendering step.

The anode (active mass ≈ 12 mg cm^−2^) was finally cut into 2 cm^2^ disks and stored in a glove box (MBraun, H_2_O and O_2_ < 0.5 ppm). All the functional tests were performed using a 2032‐type coin cell (MTI Corp.) assembled in the glove box. Metallic lithium (Sigma‐Aldrich 99.9%, thickness 0.38 mm) was used as the anode. A 1M LiPF_6_ in EC:DMC (50:50) solution (Solvionic) was the liquid electrolyte, supported by two layers of a Whatman glass fiber separator (GF/C). A small amount of fluoroethylene carbonate (FEC, 2 wt%) was used as additive.^[^ ^29^ ^]^ Coin cells with commercial graphite were also assembled in similar conditions (slurry formulation and mass loading) for the sake of comparison.

### 2.4 Physical–Chemical and Electrochemical Characterization

XRD patterns were collected on a D8 Advance diffractometer (Bruker) equipped with Cu radiation in Bragg–Brentano configuration. The diffraction patterns of the powder samples as well as the composite anodes were collected in the 10°–90° 2*θ* range, with a 0.03° step, for a total acquisition time of 3.5 h. Rietveld refinement for both powder samples and the electrodes was performed using the FullProf software.^[^ ^30^ ^]^ For the anodes, the peaks pertaining to the Cu substrate were used as an internal standard to assess relative peak shifts in the graphite patterns.

The morphological and compositional characterization of the samples was performed with a Mira3XMU microscope (TESCAN) operated at 20 kV and equipped with an EDAX–EDX analysis system. All the samples were coated with a carbon thin film using a Cressington 208 carbon coater.

Thermogravimetric analysis (TGA) was performed on a STA 8000 (Perkin Elmer) on 5–10 mg samples placed in a ceramic sample pan, running a temperature ramp between 30 and 1000 °C with 5 °C min^−1^ heating rate and an air flow of 50 mL min^−1^.

Laser granulometry measurements were carried out with LT3600Plus particle size analyzer (Linkoptik) equipped with a 20 mW, 638 nm laser, and a Venturi dry sample dispersion unit.

Raman spectra were recorded on a XploRA PLUS (HORIBA) using the following conditions: scanning range 100–1800 cm^−1^, measurement duration 20 s, accumulations 10, excitation wavelength 638 nm, laser power 1%, magnification 50×, and diffraction grating 1800 gr mm^−1^. The reported spectra were calculated as averages of a 50‐spectra mapping, covering an area of at least 80 × 80 μm^2^. Averaged spectra were then fitted using a combination of Lorentzian and Gaussian functions (3 L or 1G4L) to extract Raman peaks parameters.

ICP–OES analyses were performed by Avio 220 max (Perkin Elmer), equipped with a AVIO Glass Cyclonic Baffled spray chamber, a quartz torch, and dual backside‐illuminated charge‐coupled device detector. The quantification was carried out on acid‐digested solutions by an external standard calibration curve. ICP grade multielements standard (1000 mg L^−1^, Merck) was diluted to 0.3–0.6–2.0–5.0–9.0 mg L^−1^ and then acidified to a final concentration of 2% nitric acid (from ultrapure 65% HNO_3_, Merck). The measurements conditions were as follows: power RF 1500 W, principal gas flow 12 L min^−1^, auxiliary gas flow 0.2 L min^−1^, nebulization gas flow 0.7 L min^−1^, peristaltic pump flow 1 mL min^−1^, and frequency 500 Hz. The samples for the analysis were prepared by acid digestion of 30 mg with 3M HNO_3_ for 2 h under continuous magnetic stirring; the resulting acidic solutions were then filtered with a 0.2 μm CA syringe filter (Sartorius), diluted 1:20, and then acidified to a final concentration of 2% nitric acid (from ultrapure 65% HNO_3_, Merck).

The electrochemical tests were performed by means of potentiodynamic electrochemical impedance spectroscopy, galvanostatic cycling with potential limitation, and potentiodynamic cycling with galvanostatic acceleration (PCGA) on coin cells assembled as described earlier. A battery tester Bio‐Logic BCS‐810 was used. Cells were cycled at room temperature in the voltage range of 0.01–1.0 V. Long‐term stability tests were conducted at 0.5 C for 400 cycles (theoretical capacity of 370 mAh g^−1^). Preliminary cycles at lower current densities (namely 1st at 0.05 C, 2nd cycle at 0.01 C, 3rd–5th cycles at 0.2 C, and 6th–10th cycles at 0.25 C) were carried out as stabilization step to allow the SEI formation before the prolonged cycling test. All reported potentials refer to the Li^+^/Li couple.

## 3 Results and Discussions

### 3.1 Black Mass Characterization

**Figure 2:**
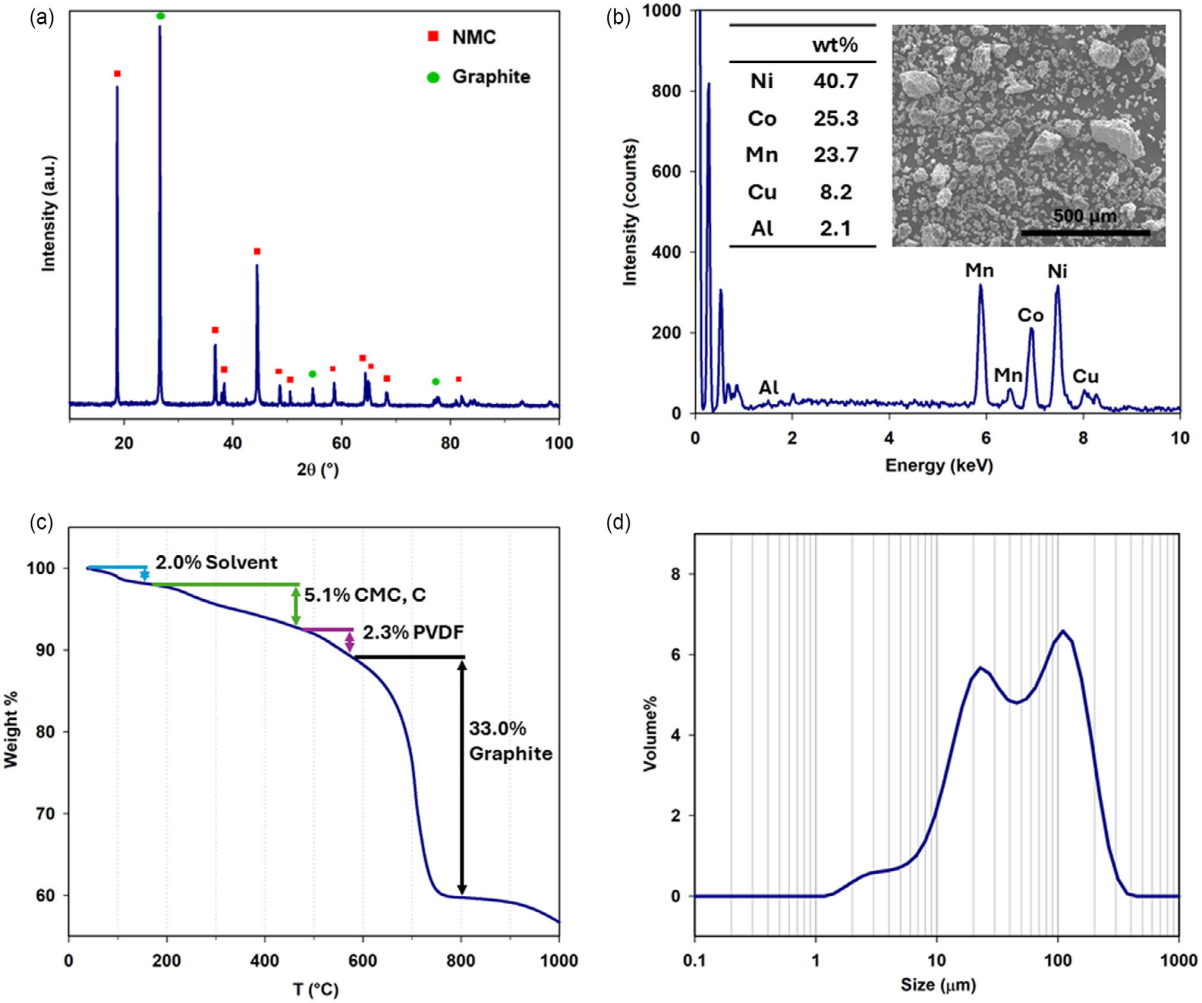
Black mass characterization. a) XRD pattern, b) EDS spectrum with element quantification and SEM image (inset), c) TGA curve, and d) particle size distribution by laser granulometry.

The BM was produced from EoL industrial LIBs modules by mechanical processing, as described in the experimental section, and characterized by means of a multitechnique approach. XRD analysis, reported in **Figure** **2**a, allowed the identification of its components, revealing the presence of graphite as AAM and lithium–nickel–manganese–cobalt oxide (NMC) as CAM. The three peaks of the XRD pattern, falling at 2*θ* = 26.6°, 54.7°, and 77.5° (green circles in Figure 2a), are related to the characteristic reflexes of the graphitic structure, while all the others, labeled with a red square, can be attributed to an NMC layered oxide structure.

The stoichiometry of the NMC‐based CAM was determined directly on the BM by means of both SEM‐EDS and ICP‐OES analysis. Figure 2b shows a large scan electron microscopy image of the BM, used for the chemical mapping, whose results are listed in the figure inset. The EDS quantification indicates an excess of Ni, when compared to the amounts of Co and Mn, which are equivalent. However, the spectra recorded on a single NMC grain (Figure S1, Supporting Information) assess a 1:1:1 stoichiometry of the cathode material (namely Ni: 32.1 at%; Mn: 33.5 at%; Co: 34.4 at%), allowing to identify the CAM chemistry as NMC111. The excess of Ni found in the BM is likely due to some unreacted NMC precursors (e.g., NiO) or even to Ni‐rich composition (e.g., NMC532) still present in the cathode, as proved by EDS analysis of the bright spherical particles, highlighted in the green inset of Figure S1, Supporting Information (Ni: 50.9 wt%, Mn: 28.4 wt%, Co: 20.7 wt%). The ratio between Mn, Co, and Ni, obtained by EDX analysis of Figure 2b and Figure S1, Supporting Information, is in very nice agreement with the ICP‐OES results, listed in Table S1, Supporting Information. The BM also includes metallic impurities coming from the current collectors, namely Cu (8.2%) and Al (2.1%), in the form of small particles as well as bulky fragments (red circle in Figure S1, Supporting Information).

TGA was carried out to estimate the composition of both anode and cathode in the BM (Figure 2c).

An initial weight loss of 2% around 100 °C is probably related to the evaporation of residual organic carbonates from the electrolyte and/or moisture. A more distinct loss of around 5% occurs between 200 and 400 °C that can be attributed to the degradation of the polymeric binders such as carboxymethylcellulose (CMC) and/or SBR, often used for the anodic material. A further small weight loss (≈2%) around 500 °C is ascribable to the degradation of polyvinylidene fluoride (PVDF), typically employed as cathodic binder. The sharpest loss, taking place between 600 and 800 °C, is associated with the decomposition of graphite in air, accounting for 33% of the total BM weight; the remaining fraction (58%) consists of cathodic oxides and metallic impurities.

Laser granulometry was finally carried out to evaluate the particle size distribution of the BM, especially for graphite. This result is important to properly design and optimise the froth flotation methodology. Mineral particles generally under the size of 50 μm, for example, are usually recovered by entrainment process more easily.^[^ ^31^ ^]^ As noticeable in Figure 2d, the BM consists of a powder with grain size lower than 200 μm. In particular, the particle size distribution exhibits three different populations: 1) a smaller one peaked at 2–3 μm, mainly associated with the CAM and possibly conductive carbon particles; 2) an intermediate one, with an average size of 20 μm, ascribable to the graphite particles; 3) a larger one around 100 μm, related to the presence of aggregates of cathodic material, likely favored by the presence of polymeric binders and fragments of current collectors (as confirmed by SEM–EDS analysis).

### 3.2 Graphite Recovery and Regeneration

The separation of the cathodic and anodic fractions of the BM was performed through froth flotation, a well‐known technique deeply used for the recovery of minerals. As stated before, such separation technology exploits the wettability and density contrast between the surface of different mineral particles (in this work graphite and NMC), with the help of air bubbles introduced in the system. In a flotation pulp, in fact, the hydrophobic particles attach to air bubbles forming aggregates that float, are dragged to the top and trapped in the froth, whereas the hydrophilic particles are collected at the bottom of the cell as tailings fraction.

Graphite and NMC were separated from the BM as detailed in the experimental section. Paraffin oil (collector) and pine oil (frother) with a dosage of 300 g_agent_ t^−1^ were added to the BM after the attritioning step carried out by means of high shear mixing in water. It was recently demonstrated that this pre‐treatment favours the removal of residual binder coating, activating the particle surface and enhancing the dispersion capability of graphite in water. This results in a more efficient flotation and higher quality of the recovered materials by significantly reducing the amount of NMC going to the overflow product, that is usually promoted by true flotation and entrainment.^[^ ^24^ ^]^

One single fraction of the overflowing froth was collected at flotation time *t* = 300 s, corresponding to more than 38% of the pristine BM with a grade of Carbon material (*C%*) higher that 88% (see **Table** **1**) and an overall recovery yield of >96%. This value is in very nice agreement with, or even higher than, what obtained in case of graphite separation from BM via froth flotation with kerosene and/or MIBC.^[^ ^19^, ^20^, ^24^, ^32^ ^]^ The tailings, containing the CAM, was also isolated by filtration and characterized by TGA to quantify the residual impurities of graphite, which resulted to be 2 wt% (see Figure S2, Supporting Information).

**Table 1:** d‐spacing value for (002) crystal plane, peak width (FWHM), and cell parameters (a, b, and c) calculated by Rietveld refinement, Raman *I*_D_/*I*_G_ value, average granulometric size (D50), tap density (TD), and carbon content (C%) for graphite powder after flotation (G‐PF), leaching (G‐PL), and thermal treatment (G‐PT).

Sample	*d*_002_ [Å]	FWHM [°]	*a, b* [Å]	*c* [Å]	Raman *I*_D_/*I*_G_	*D*_50_ [μm]	TD [g cm^−3^]	*C* [%]
G‐PF	3.3595(4)	0.166	2.4643(2)	6.7190(8)	0.229(2)	16.3(5)	0.91	88.4
G‐PL	3.3605(3)	0.239	2.4635(1)	6.7210(5)	0.234(2)	16.7(5)	0.90	98.2
G‐PT	3.3604(3)	0.239	2.4634(1)	6.7207(5)	0.435(5)	23.6(7)	0.86	99.6

The recovered graphite (labelled in the following as graphite post‐flotation, G‐PF) underwent chemical purification to remove the residual impurities coming from the CAM and the solid electrolyte interface. This refining step was carried out through mild acid leaching, at low temperature with green chemicals. As described in section 2.2, a 0.5 M solution of citric acid was used at 75 °C for 2 h to obtain high‐purity material for a potential re‐use in the remanufacturing of new cells. The purified graphite is labelled in the following as graphite post‐leaching, G‐PL.

After the chemical purification, the recovered graphite (G‐PL) was thermally treated at 900 °C under Ar flow for 2 h, aiming to further increase the material purity by removing degradation byproducts as well as healing damages of the particle morphology and crystal structure induced by prolonged ageing.^[^ ^33^ ^]^ The treated material (labelled as graphite post‐treatment, G‐PT) together with G‐PF and G‐PL were characterized by a multi‐technique approach and the results discussed in the following sections.

### 3.3 Physical‐Chemical Characterization of the Recycled Graphite

**Figure 3:**
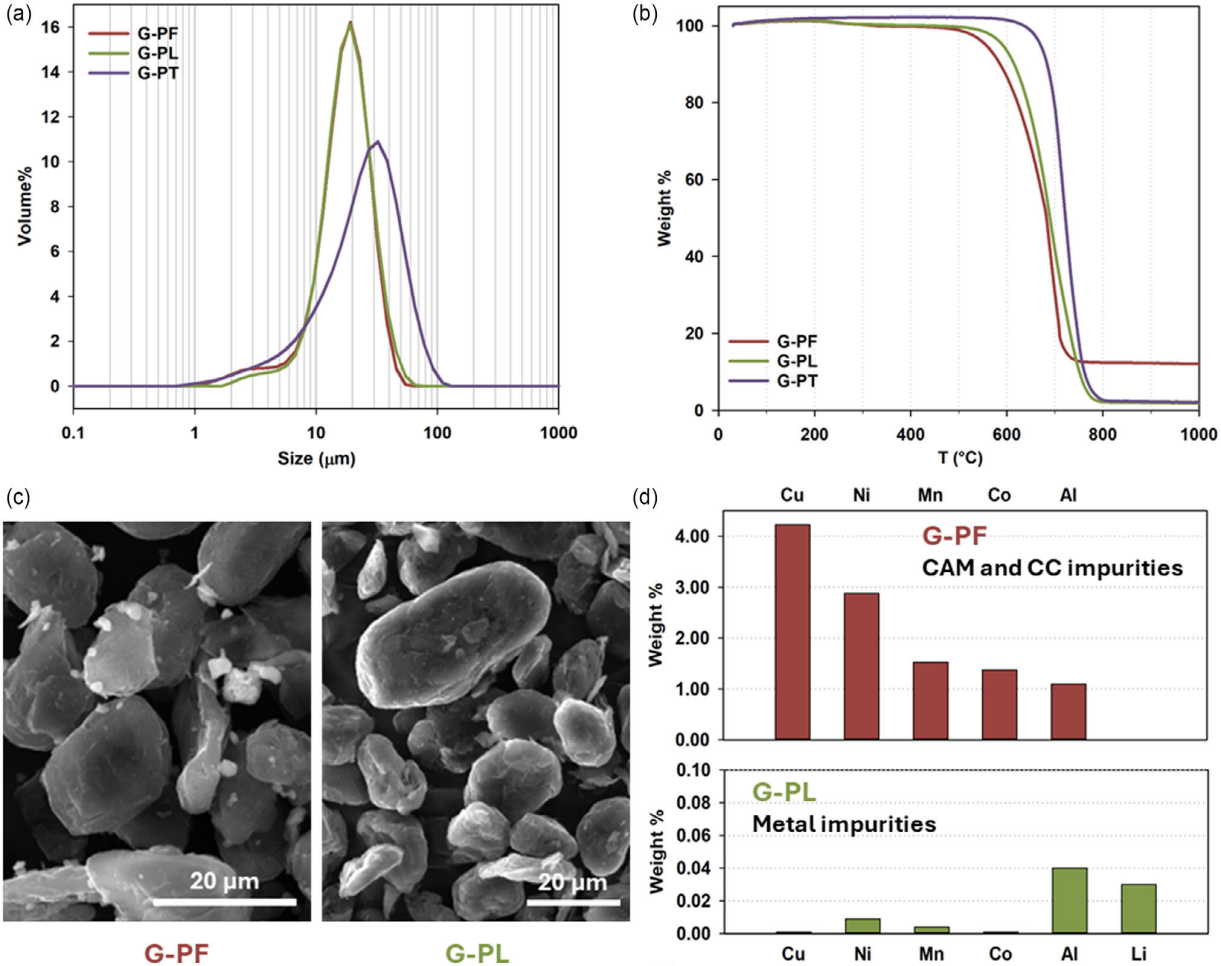
Morphological and compositional characterization of recovered graphite after flotation, leaching, and thermal treatment. a) Size distribution and b) TGA curves of G‐PF (red), G‐PL (green), and G‐PT (violet); c) SEM images of G‐PF (left) and G‐PL (right); d) wt% of metals in G‐PF by EDS (red, top) and in G‐PL by ICP–OES (green, bottom).

The tap density and particle size distribution of all the samples (G‐PF, G‐PL, G‐PT), reported in Table 1, are in nice agreement with the commercial reference and the values usually obtained for the graphite recovered by means of froth flotation or delamination processes.^[^ ^15^, ^19^ ^]^ The chemical purification does not substantially change the graphite tap density with respect to the recovered material after flotation. Instead, a lower value is determined after the thermal treatment, in agreement with the literature.^[^ ^15^ ^]^ A similar trend is observed for the particle size, as reported in Table 1 and **Figure** **3**a. In this case, G‐PT shows slightly larger particles, likely due to thermally induced aggregation; moreover, the thermal treatment enhances the sample purity as confirmed by the significant decrease of the peak of the size distribution (Figure 3a) related to smaller objects (2–3 μm), generated by organic residuals (e.g., binders, flotation reagents or leaching acid leftovers). This is further confirmed by thermogravimetric analysis, reported in Figure 3b, where no weight loss at 300 °C is visible for G‐PT sample. TGA measurements also allowed to determine the Carbon content (*C%*) in the recovered materials. As expected, the *C%* value increases thanks to the purification step, exceeding 99.5% after the thermal treatment at 900 °C.

The graphite specific surface area (from BET analysis) was measured only for the thermally treated sample (G‐PT) and was found to be 6.4 m^2^ g^−1^.

Figure 3c shows the SEM images of G‐PF and G‐PL samples, revealing a potato‐shape morphology with particle size of dozens of micrometres, in line with the laser granulometry output. The G‐PF image also reveals the presence of BM impurities as brighter grains scattered around graphite (Figure 3c, left), which in turn disappear in the case of G‐PL (Figure 3c, right) due to the effective chemical purification by means of citric acid. EDS analysis allowed to determine that these impurities are ascribable not only to the cathodic material, but also to the current collectors (CC), since Cu (4.2%) and Al (1.1%) were found, together with Ni (2.9%), Mn (1.5%) and Co (1.4%), as summarized in Figure 3d (top) and Figure S3, Supporting Information (reporting the entire EDS spectrum). By comparing these results (Figure 3d, red top) with the chemical analysis carried out on the pristine BM (Figure 2b), it is possible to conclude that the froth flotation is more effective to separate the CAM transition metals from the graphite than Cu and Al. However, the removal of metallic impurities (including Li) by acid leaching was successful, as confirmed by ICP–OES analysis performed on G‐PL. Figure 3d (bottom) reports the metals content found in the leached graphite (G‐PL), namely <0.05 wt% for Al and Li and <0.01 wt% for Cu, Ni, Mn and Co, demonstrating a very high purity degree of the recovered sample. Such values are at least one magnitude order lower than those reported in literature in case of graphite recovered by froth floatation and refined through harsher washing.^[^ ^11^, ^14^, ^20^ ^]^

**Figure 4:**
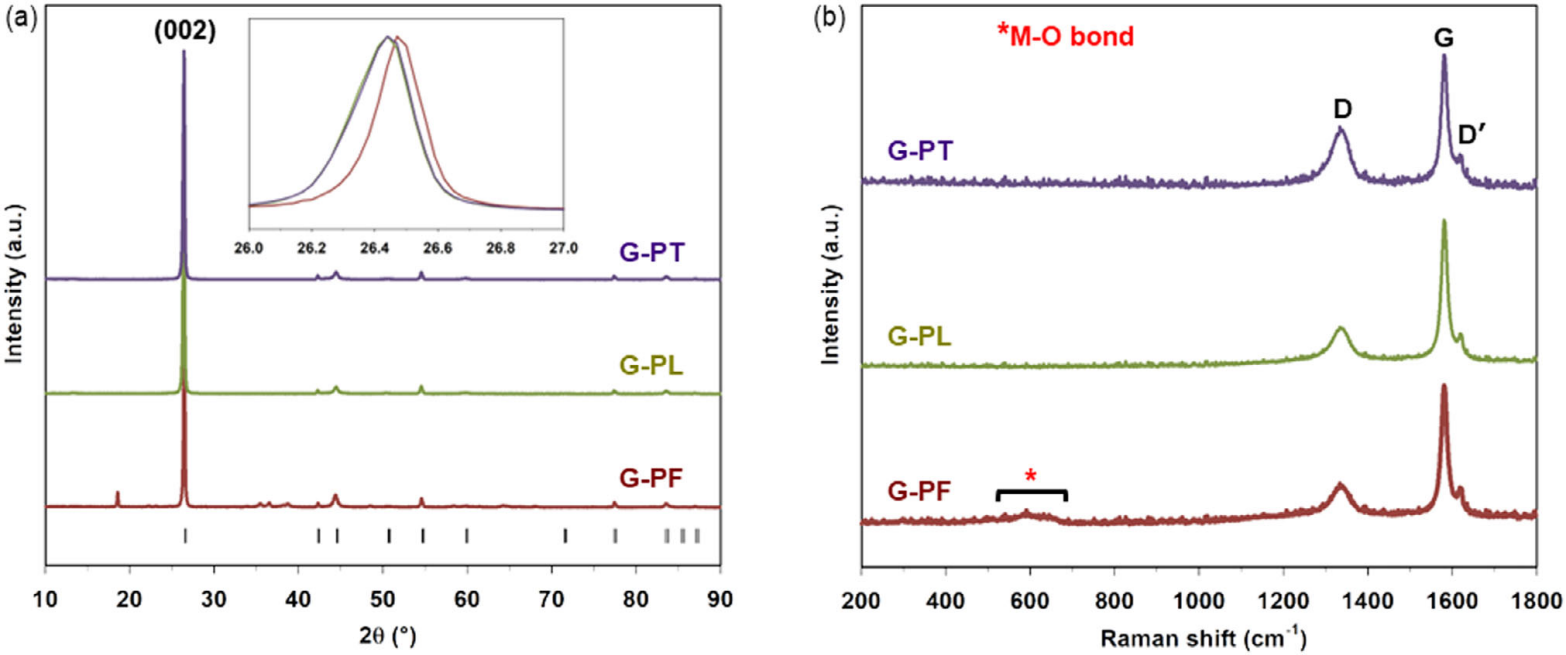
Structural characterization of recovered graphite after flotation, leaching, and thermal treatment. a) XRD patterns and b) average Raman spectra of G‐PF (red, bottom), G‐PL (green, middle), and G‐PT (violet, top). Black bars under XRD patterns indicate Bragg positions for graphite; inset shows an enlargement of the main peak.

**Figure** **4**a shows the XRPD patterns of the graphite samples collected after each recycling step, namely G‐PF, G‐PL and G‐PT. All the patterns show a preferential orientation along the (0 0 L) diffraction planes, promoted by the sample preparation procedure for the measurement in Bragg‐Brentano geometry. G‐PF contains spurious peaks associated to the presence of the NMC cathodic impurities. After the leaching process, the purity of the sample is greatly improved and all the peaks in G‐PL can be indexed in the *P*6_3_ *mc* space group, according to the graphite structure.^[^ ^34^ ^]^ However, a shift in the position of the main graphite peak, related to the (0 0 2) plane, is evident with respect to G‐PF (inset of Figure 4a). In particular, the calculated *d*‐spacing for this plane slightly increases after the leaching step (see Table 1). It is also relevant to note that the main peak is broadened after the leaching treatment, with a calculated full width at half maximum (FWHM) changing from 0.166° for G‐PF to 0.239° for G‐PL. The broadening of the peak is indicative of a larger distribution in the distances between graphitic sheets, which in turn suggests an increase of the degree of disorder.

This result is confirmed by Raman spectroscopy, which can usually give additional information on the graphite crystal structure, graphitization degree and near‐surface region of the particles. Figure 4b compares the spectra collected for the material throughout the entire recycling process, namely post‐flotation, post‐leaching and after thermal treatment. Contrary to the purified materials, the Raman spectrum of G‐PF presents an additional peak around 600 cm^−1^ ascribable to the vibrational mode of the metal‐oxygen (M*—*O) bonds, suggesting the presence of the CAM impurities, as expected. In each investigated sample, the spectra show the typical vibration modes of graphite, namely *G*, *D* and *D’*, falling respectively around 1580, 1350, and 1620 cm^−1^. As known, the *D*‐band corresponds to the formation of graphite structural defects. A slight increase of the *D* peak is evident in case of thermally treated graphite. This trend agrees with the *I*_D_ */I*_G_ value (i.e., the intensity ratio related to the *D* and *G* modes), calculated from the Raman spectra (Table 1), that directly correlates to the degree of disorder of a carbon‐based material.^[^ ^35^ ^]^ Indeed, the value of *I*_D_ */I*_G_ remains almost constant for G‐PF and G‐PL (i.e. 0.229 and 0.234, respectively), suggesting that the chemical purification does not affect the graphitization degree of the recovered material. Conversely, *I*_*D*_ */I*_*G*_ increases to 0.435 for G‐PT, suggesting an enhanced disorder after the thermal treatment, which is in very good agreement with the information obtained from XRD. This value likely accounts for the presence of an amorphous carbon phase generated by the thermal annealing, which could positively contribute to the anodic material conductivity.^[^ ^33^ ^]^ However, values of *I*_D_ */I*_G_ ranging between 0.2–0.5 are characteristic as index of graphitic ordering for commercial graphites, even in absence of any heat treatment.^[^ ^36^ ^]^

### 3.4 Validation of the Recycling Process by Electrochemical Tests on Half Cells

The suitability of the proposed recovery and regeneration process was validated by using the fully recycled and purified graphite (G‐PT) as anode in Li half cells (labelled as R‐G). The functional performances were then compared to those obtained in similar configuration including commercial graphite as baseline (labelled as C‐G). Both the electrodes were characterized from a structural and morphological point of view both before and after prolonged galvanostatic cycling to evaluate the stability of the recycled graphite with respect to the benchmark. Figure S4a–b and S5a‐b, Supporting Information show the SEM images (surface and cross‐section, respectively) collected for the regenerated and commercial graphite anodes, evidencing similar morphology and comparable particle size. Figure 4c–d reports the XRD patterns of the two non‐cycled electrodes. The peaks due to the Cu substrate are clearly visible, while it is evident that all the patterns show a high degree of preferential orientation along the (0 0 L) plane, higher than that presented by the powder samples in Figure 4a.

**Table** **2** reports the cell parameters and the calculated *d*‐spacing for R‐G and C‐G before cycling, obtained by the refined patterns shown in Figure S6, Supporting Information. In general, *d*‐spacing values for the anodes are lower compared to those calculated for the powder samples (see Table 1); this is reasonably due to the electrode preparation procedure, which implies the use of film calendaring. The results summarized in Table 2 show that the two samples (R‐G and C‐G), before cycling, are characterized by very similar average distances between graphite layers along the *c* axis. In contrast, a comparison between the FWHM values suggests that the regenerated sample is characterized by a wider distribution of *d*‐spacing than in the commercial sample, corresponding to a larger value of FWHM. It is interesting to note that the FWHM for the regenerated graphite anode is significantly reduced compared to the corresponding powder samples (Table 1), suggesting that the preparation process produces a higher degree of orientation in the sample in consequence of the calendaring step.

**Table 2:** d‐spacing value for (002) crystal plane, peak width (FWHM), and cell parameters (a, b and c) calculated by Rietveld refinement for the anodes with regenerated graphite (R‐G) and commercial graphite (C‐G) before and after the prolonged cycling.

Anode	*d*_002_ [Å]	FWHM [°]	*a, b* [Å]	*c* [Å]
R‐G	3.3538(3)	0.151	2.4631(1)	6.7076(6)
R‐G—400th cycle	3.3561(2)	0.200	2.4629(9)	6.7121(3)
C‐G	3.3542(3)	0.123	2.4627(1)	6.7084(5)
C‐G—400th cycle	3.3538(4)	0.153	2.4627(4)	6.7077(7)

**Figure 5:**
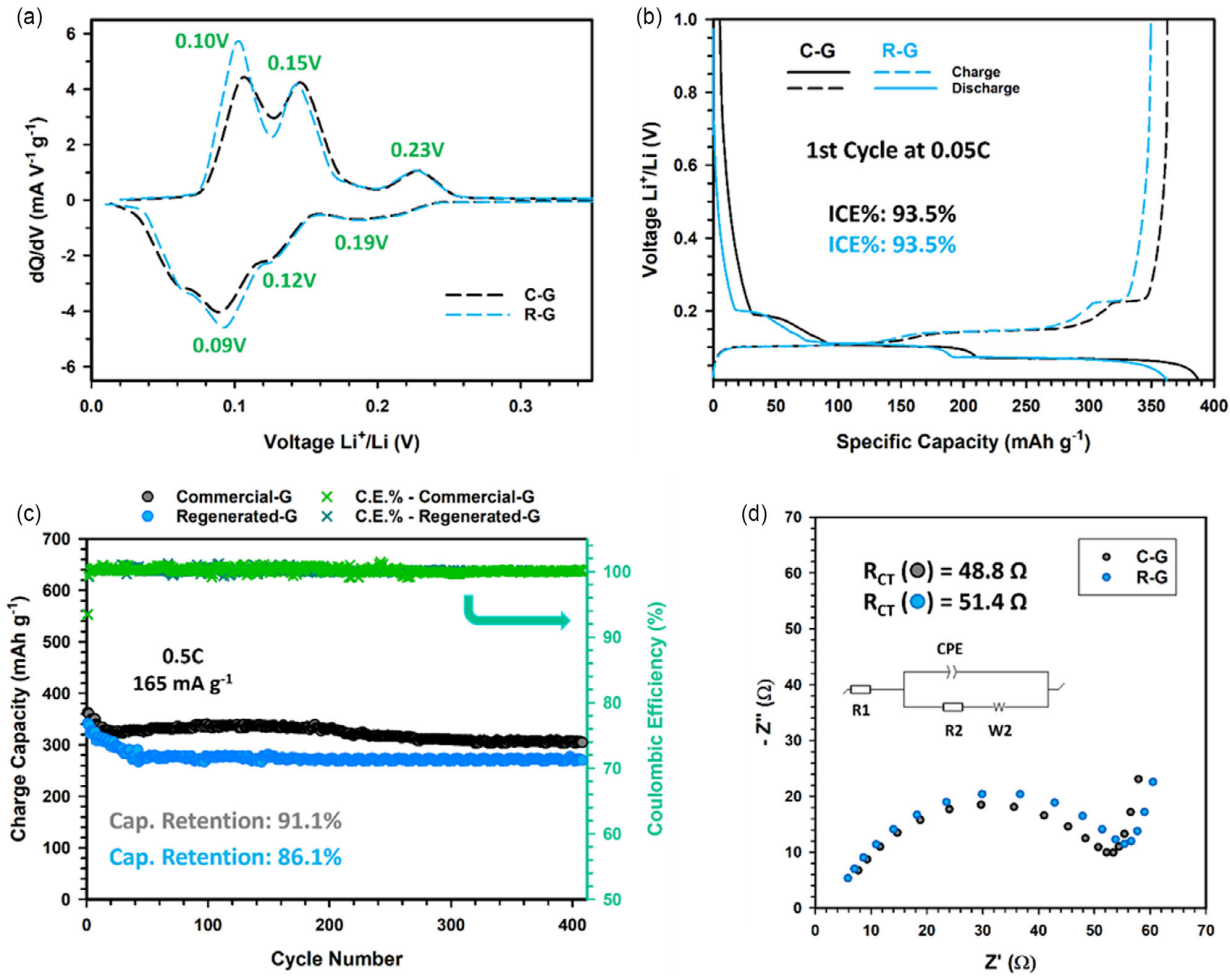
Electrochemical characterization of regenerated graphite (R‐G, light blue) and commercial graphite (C‐G, black). a) Differential capacitance (dQ/dV) plots of the second cycle between 0.01 and 0.35 V; b) voltage profile of the first cycle at 0.05 C (I: 16.5 mA g^−1^) and c) long‐term cycling test reporting the discharge capacity and coulombic efficiency; d) EIS plots collected after 400 cycles performed at 0.5 C.

**Figure** **5**a compares the differential capacitance plots, obtained from the second cycle of *PCGA* experiments on cells including the regenerated (R‐G) and commercial graphite (C‐G), as described in the experimental section. The plots referred to the 1st and 3rd cycle are reported in Figure S7a,b, Supporting Information. The curves are almost overlapping and show high reversibility of the electrochemical phenomena in both cases.

The *dQ/dV* profiles exhibit the characteristic peaks that are indicative of the lithium intercalation/deintercalation process. The three peaks at 0.23, 0.15, and 0.1 V (vs. Li/Li^+^) correspond to the Li^+^ intercalation, while the peaks at 0.09, 0.12, and 0.19 V refer to the Li^+^ deintercalation.^[^ ^37^ ^]^ The same phenomena are also clearly visible in the charge–discharge profiles of the initial cycle performed at 0.05 C, as demonstrated in Figure 5b. The presence of well‐defined multiple plateaux is a distinctive feature of the expected phase transitions that occur during the lithiation and delithiation of graphite.^[^ ^38^, ^39^ ^]^

The R‐G anode exhibits a specific capacity of 341 mAh g^−1^, very close to the value delivered by the C‐G cell (360 mAh g^−1^), and an excellent initial Coulombic efficiency (ICE) of 93.5%, in perfect agreement with the baseline or other commercial systems,^[^ ^40^ ^]^ and even higher than what reported in literature in case of recycled graphite reused as anode for LIBs.^[^ ^11^, ^14^ ^]^

The results of the long‐term cycling test, performed at 0.5 C (165 mA g^−1^) for 400 cycles, are reported in Figure 5c. Overall, the regenerated graphite exhibits electrochemical performances that are well comparable to those of the commercial one both in terms of cycling stability and coulombic efficiency (see Figure S7c,d, Supporting Information). The R‐G and C‐G anodes, in fact, show capacity retention higher than 91% and 86%, respectively, achieving discharge capacity of 270 mAh g^−1^ (R‐G) and 304 mAh g^−1^ (C‐G) after 400 cycles at 0.5 C.

Figure 5d compares the electrochemical impedance spectra collected at the end of the long‐term cycling test for R‐G and C‐G anodes. The spectra were fitted by means of the equivalent circuit shown in the figure inset to determine the interfacial resistance of the cell, *R*_CT_. Only slight difference of *R*_CT_ values (e.g. 48.8 Ω for C‐G and 51.4 Ω for R‐G) is determined from the Nyquist plots, suggesting that the regenerated and commercial graphite exhibits similar behavior at anode/electrolyte interface level.

**Figure 6:**
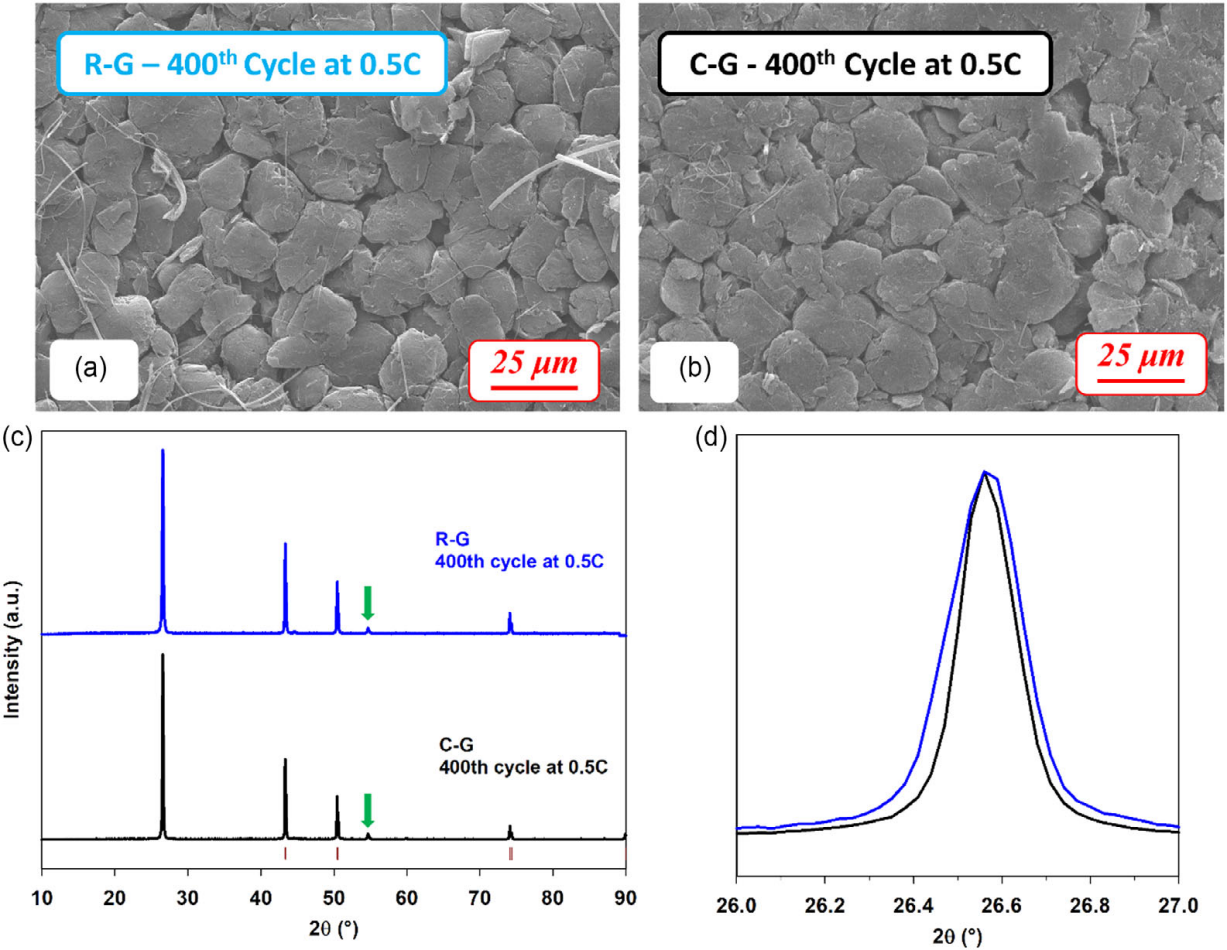
SEM images of the surface of the anodes with a) regenerated graphite, R‐G, and b) commercial graphite, C‐G after 400 cycles performed at 0.5 C (*I* = 165 mA g^−1^). c) XRD patterns of R‐G (blue) and C‐G (black) after cycling and d) enlargement of the main graphite peaks. Red bars under the patterns indicate Bragg positions for Cu substrate.

SEM (**Figure** **6**a–b) and XRD characterization (Figure 6c–d) of graphite after the prolonged cycling process confirms the material structural integrity, both for the commercial and regenerated sample (see also Figure S5c–d, Supporting Information showing the cross‐section SEM images). More specifically, the anodes did not undergo any substantial morphological modification after 400 cycles at 0.5 C (Figure 6a,b and S5c‐d, Supporting Information). XRD patterns for R‐G and C‐G, reported in Figure 6c, evidence that the degree of preferential orientation and order is partially reduced, as suggested by the appearance of small peaks, in addition to the characteristic (0 0 L) reflections, such as the (1 0 1) one at 2*θ* = 44.5° (green arrow). The partial loss of orientation is consistent with the resulting increase of FWHM values upon cycling (see Table 2) and is more evident for the regenerated graphite.

## 4 Conclusions

This work demonstrates the effective recovery of graphite with yield higher than 96% from EoL LIBs BM by means of a low‐impact froth flotation based on green chemicals. After mild chemical purification followed by thermal treatment, high‐quality graphite was obtained with carbon content of 99.6%. Graphite was recovered while preserving excellent morphology and microstructure. The proposed direct recycling process was validated through a closed‐loop strategy from EoL cells to new cells, reusing the recovered material as regenerated anode. The electrochemical properties of the recycled graphite were comparable to those obtained with a commercially available graphite as baseline, achieving an initial coulombic efficiency of 93.5% and allowing an excellent cycling stability for more than 400 cycles with capacity retention of about 86%.

While there is still a plenty of room for improvement and optimization, these results outline the great potential of the direct recycling to gain almost all the spent graphite from EoL‐LIBs that is crucial to ensure a reliable supply of this valuable material. The proposed solutions can contribute to support the development of direct recycling technologies to secure their scale‐up at industrial level.

## Conflict of Interest

The authors declare no conflict of interest.

## Supporting information

Supplementary Material

## Data Availability

The data that support the findings of this study are available from the corresponding author upon reasonable request.
