# Supplementary material for: A Green Process for Effective Direct Recycling and Reuse of Graphite from End‐of‐Life Li‐Ion Batteries Black Mass
Source: ChemSusChem. 2025 Jul 8;18(17):e202500550. doi: 10.1002/cssc.202500550 (PMC12404021; doi:10.1002/cssc.202500550)
Supplement: Supplementary file 1 — Supplementary Material [file CSSC-18-e202500550-s001.pdf]

Supporting Information

A green process for effective direct recycling and reuse of graphite from End-of-Life Li-ion batteries black mass

L. De Vita[1,2], D. Callegari[1,2], A. Bianchi[1], C. Tealdi[1,2], N. Zucca[3], P. Galinetto[3], M. Colledani[4] and E. Quartarone[1,2 *]

[1]*Department of Chemistry, University of Pavia, Via Taramelli 12 27100 Pavia, Italy*

[2]*Consorzio Interuniversitario per la Scienza e la Tecnologia dei Materiali INSTM, Via Giusti 9 50121 Firenze, Italy*

[3]*Department of Physics, University of Pavia, Via Bassi 6 27100 Pavia, Italy*

[4]*Department of Mechanics, Polytechnic University of Milan, Via Privata G. La Masa 1 20156 Milan, Italy*

*Corresponding author: eliana.quartarone@unipv.it

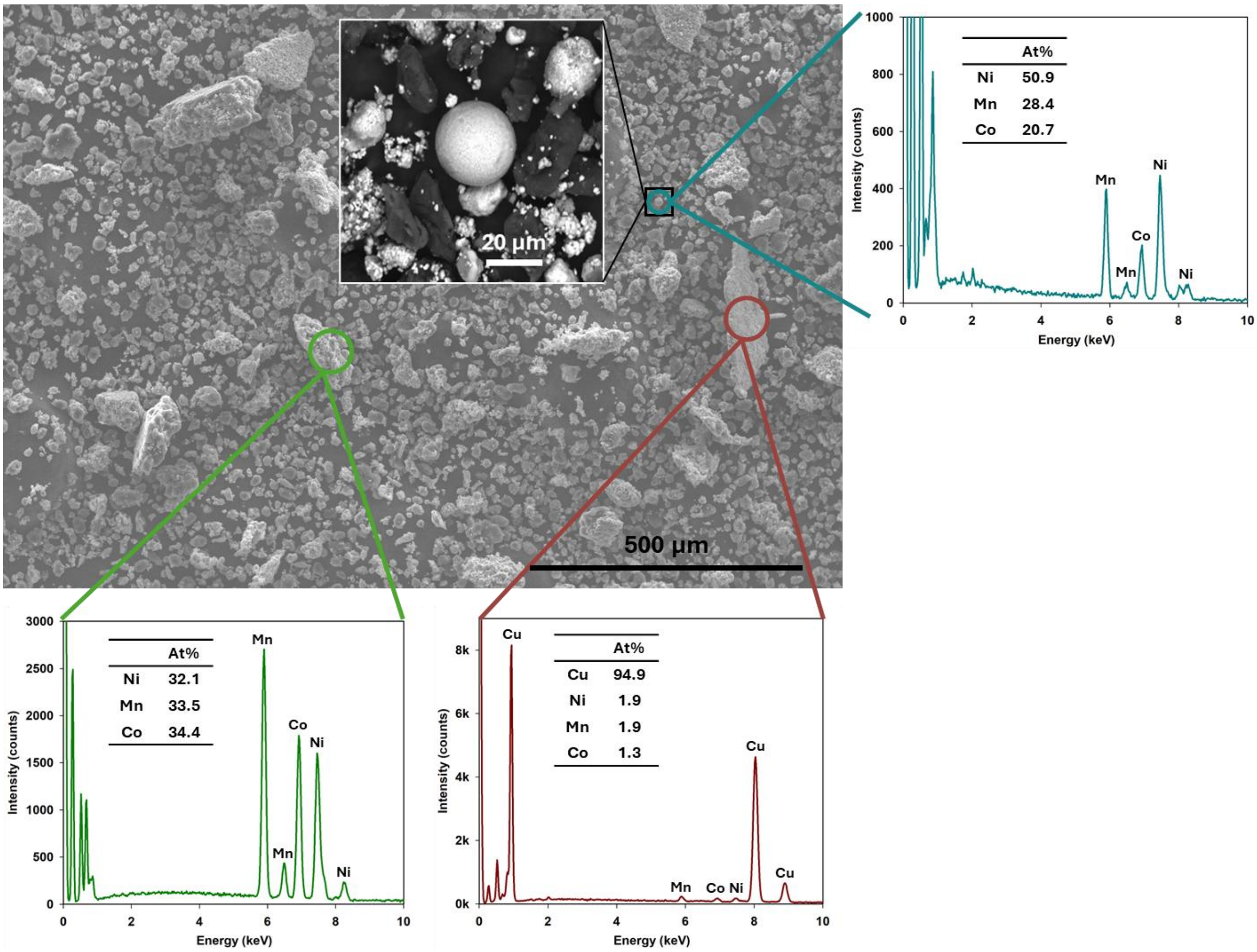

Figure S1 *SEM image of the black mass and EDS point spectra with element quantification on different CAM particles (green and blue) and on a Cu current collector fragment (red); inset, magnification of a Ni-rich particle.*

	**w%**		**w%**
**Ni**	**17.3**	**Li**	**3.7**
**Co**	**10.9**	**Cu**	**1.4**
**Mn**	**10.3**	**Al**	**<0.1**

Table S1: *ICP-OES analysis performed on the black mass*

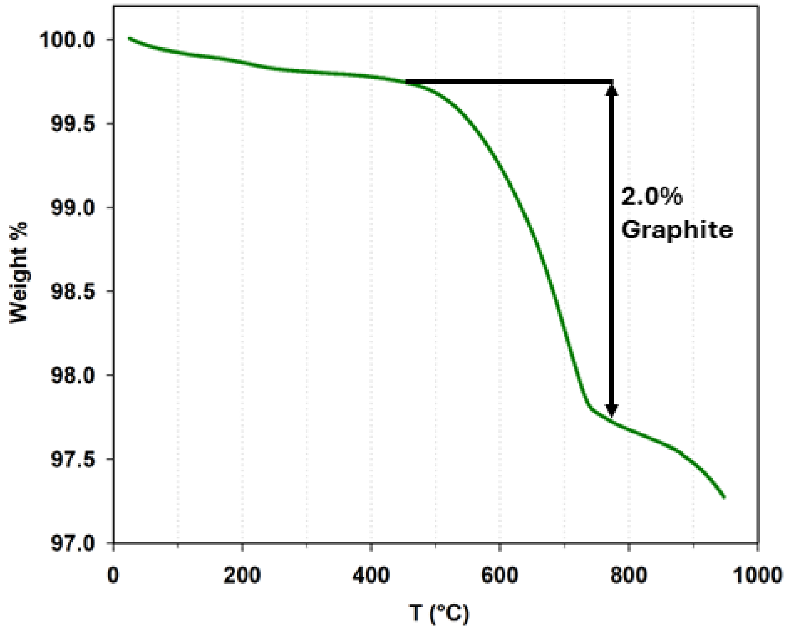

Figure S2 *TGA plot of the CAM (NMC111) recovered as underflow from the froth flotation process carried out on the black mass*

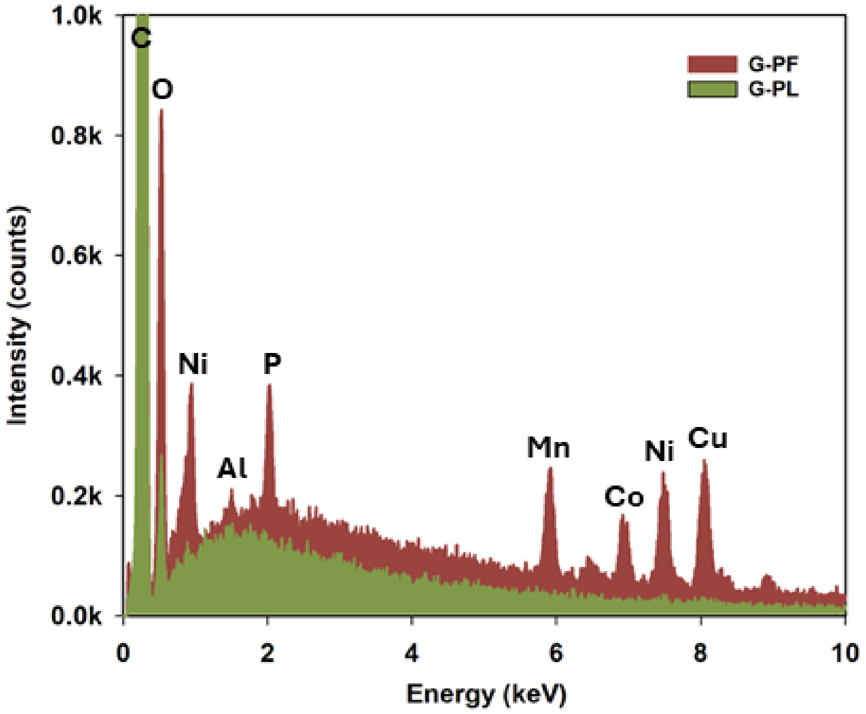

Figure S3 *EDS spectra of graphite after flotation (G-PF, red) and after leaching (G-PL, green).*

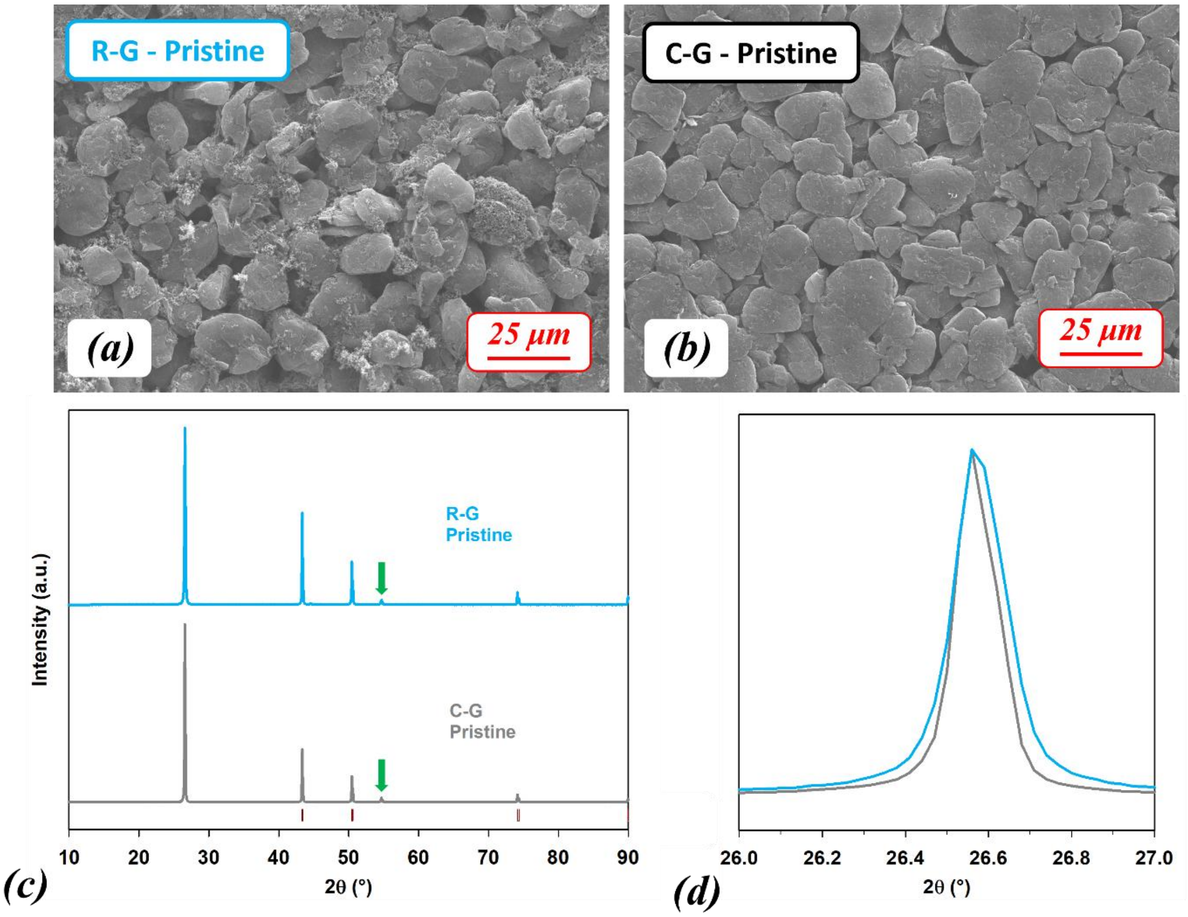

Figure S4. *Pre-cycling SEM images of the surface of (a) R-G and (b) C-G based anodes. (c) XRD patterns of the anodes with regenerated graphite, R-G (in blue), and commercial graphite, C-G (in black) before electrochemical test; (d) Enlargements of the main graphite peaks. Red bars under the patterns indicate Bragg positions for Cu substrate.*

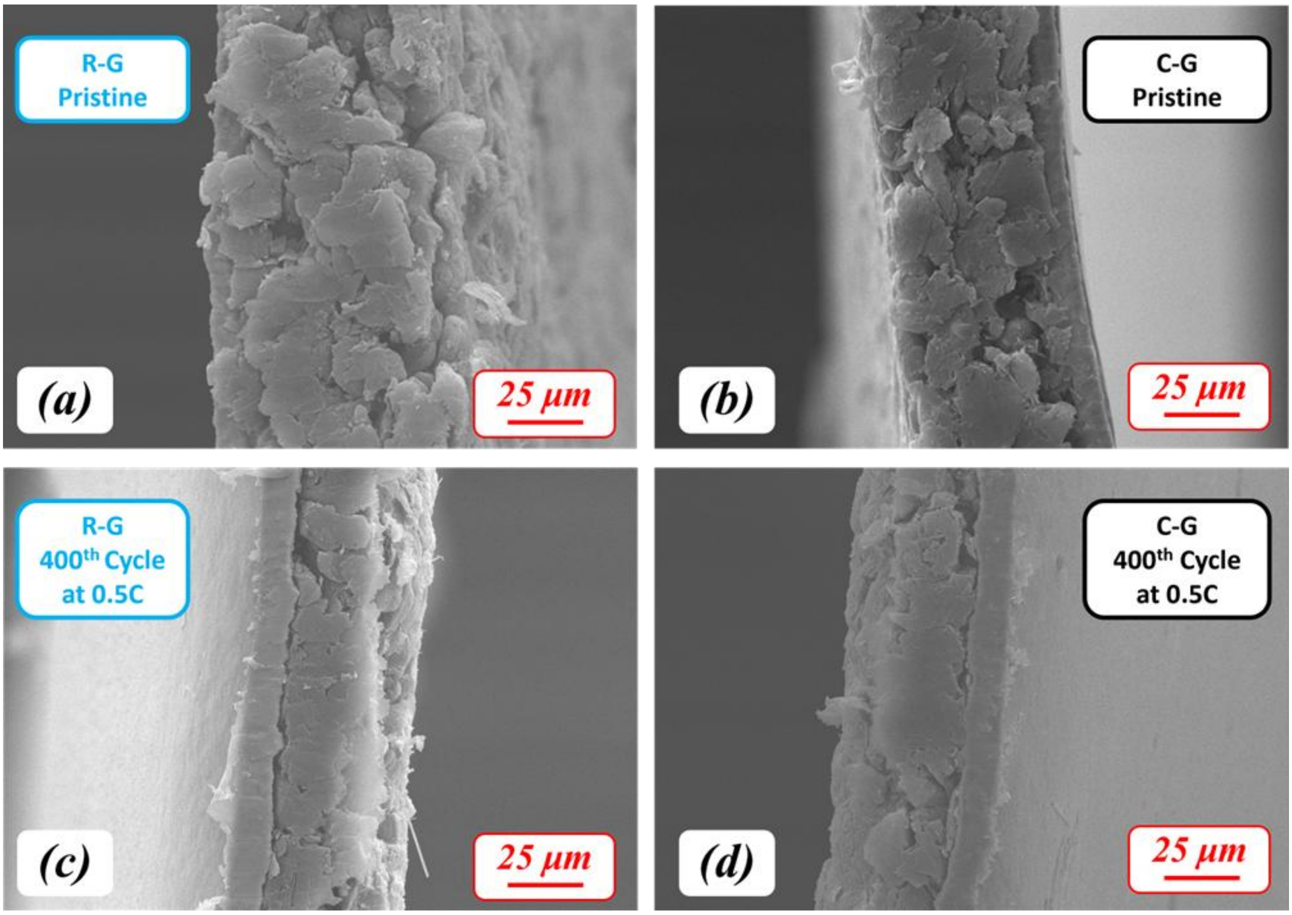

Figure S5 *Cross-Section SEM images of the anode before (a, b) and after (c,d) the long-term stability test for R-G and C-G, respectively.*

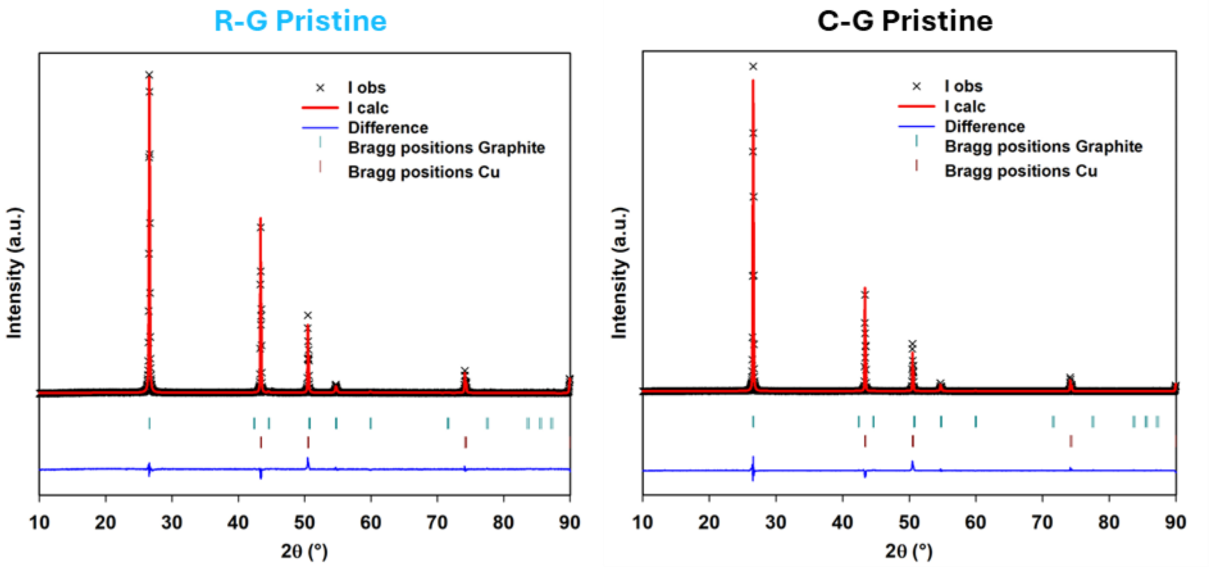

Figure S6 *Rietveld refined XRD patterns of the anodes with regenerated graphite (R-G, left) and commercial graphite (C-G, right). Green and red bars indicate Bragg positions for graphite and Cu, respectively.*

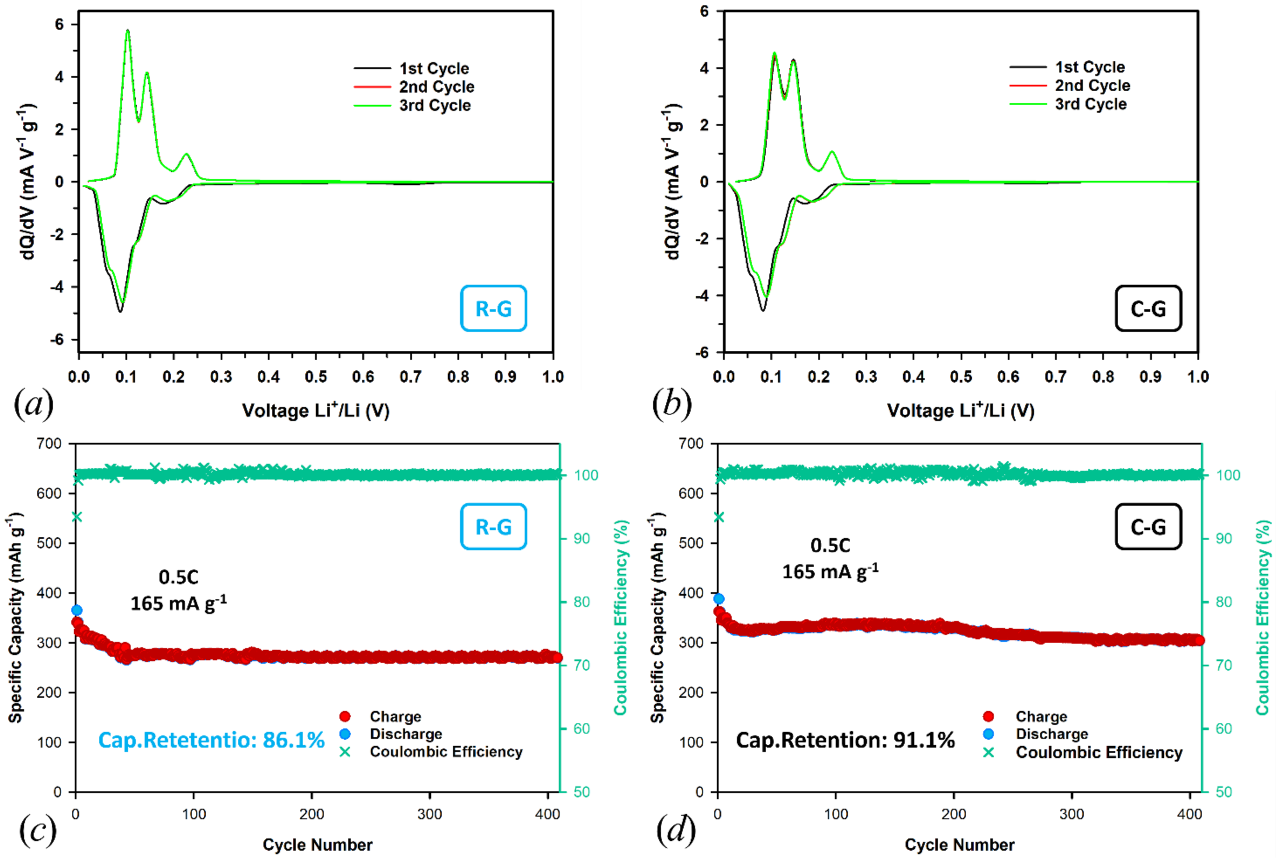

Figure S7 *Differential capacitance (dQ/dV) plots in the voltage range between 0.01V – 1.0V for (a) Regenerated Graphite and (b) Commercial Graphite. (c, d) Long-term cycling test reporting the specific capacity and coulombic efficiency of R-G and C-G, respectively. The first cycle was performed at 0.05C, the second at 0.01C, the next five cycles at 0.2C, followed by an additional five cycles at 0.25C, before increasing the C-rate to 0.5C for 400 cycles.*
